# Chikusetsusaponin IVa targeted YAP as an inhibitor to attenuate liver fibrosis and hepatic stellate cell activation

**DOI:** 10.1186/s13020-025-01090-5

**Published:** 2025-03-17

**Authors:** Kai Gao, Wei Zhang, Dong Xu, Meina Zhao, Xingru Tao, Yunyang Lu, Jingwen Wang

**Affiliations:** 1 Department of Pharmacy, Xijing Hospital, Fourth Military Medical University, Xi’an, 710032 China; 2 Department of Chinese Materia Medica and Natural Medicines, School of Pharmacy, Fourth Military Medical University, Xi’an, 710032 China; 3 Research Institution, Xijing Hospital, Fourth Military Medical University, Xi’an, China

**Keywords:** Liver fibrosis, Hepatic stellate cells, Chikusetsusaponin Iva, SPR, YAP

## Abstract

**Background:**

Liver fibrosis is a representative scarring response that can ultimately lead to liver cancer. However, relevant antifibrotic drugs for the effective treatment of liver fibrosis in humans have not yet been identified. Chikusetsusaponin IVa (CS-IVa) is derived from natural products and exhibits multiple biological activities; however, its efficacy and potential mechanism of action against liver fibrosis remains unclear.

**Purpose:**

This study aimed to examine the antifibrotic properties and potential mechanisms of action of CS-IVa.

**Methods:**

We constructed two mature mouse models (CCl_4_ challenge and bile duct ligation) to evaluate the antifibrotic properties of CS-IVa in vivo. Proteomics analysis and transforming growth factor β1 (TGF-β1)-activated LX-2 cells were used to elucidate the potential effects and mechanisms. Molecular docking, surface plasmon resonance (SPR), and cellular thermal shift assay (CETSA) were used to detect the affinity and binding between CS-IVa and its target.

**Results:**

We found that CS-IVa significantly alleviated liver fibrosis and injury by downregulating yes-associated protein (YAP) and tafazzin (TAZ) expression. In an in vitro model, CS-IVa suppressed TGF-β1-induced hepatic stellate cell (HSC) activation, as well as the mRNA and protein expression of COL1A1, α-SMA, YAP, and TAZ. Moreover, specific knockdown or inhibition of YAP did not enhance the suppressive effect of CS-IVa on HSC activation or fibrosis-associated protein expression. Molecular docking, SPR, and CETSA showed that CS-IVa could directly bind to YAP.

**Conclusion:**

These findings demonstrated that the administration of CS-IVa effectively alleviated liver fibrosis by suppressing the YAP/TAZ pathways. In addition, CS-IVa could directly bind to YAP and act as a YAP inhibitor.

## Introduction

Liver fibrosis is attributed to an excessive reparative response triggered by various chronic hepatic injuries (such as viral hepatitis, alcoholic hepatitis, metabolic-associated steatohepatitis, and autoimmune liver disease), marked by an overabundance of extracellular matrix (ECM) deposition and the generation of fibrous scars, which, in the absence of timely intervention, could promote the development of liver cirrhosis or hepatocellular carcinoma [1]. The pathogenesis of liver fibrosis is significantly influenced by hepatic stellate cell (HSC) activation. Quiescent HSCs store vitamin A and maintain ECM homeostasis. During inflammation or other stimulus-induced liver damage, HSCs are activated and differentiate into myofibroblasts, leading to the depletion of their vitamin A reserves and substantial production of collagen and ECM [2]. The effective inhibition of HSC activation can contribute to liver fibrosis treatment [3]. However, the current therapeutic options for liver fibrosis are limited [4]. Therefore, the development of novel antifibrotic drug candidates is of paramount importance.

The activation of the Hippo pathway is highly associated with the progression of liver fibrosis [5, 6]. The Yes-associated protein (YAP) is activated during the initial stages of HSC activation and subsequently translocates to the nucleus, thereby facilitating liver fibrosis by regulating target genes at a transcriptional level [7]. According to recent research findings, deactivation of Hippo pathway kinases results in nuclear translocation of YAP/TAZ protein and subsequent interaction with TEAD1-4 transcription factors, leading to enhanced activation of target genes such as *Ctgf* (connective tissue growth factor) and *Ankrd1* (cardiac ankyrin repeat protein), which contribute to the exacerbation of hepatic fibrosis [8]. According to a previous study, YAP-S127A, which is expressed in hepatocytes, exacerbates CCl_4_-induced liver fibrosis, whereas the deficiency of YAP in hepatocytes exerts the opposite effects [9]. Another research supported these findings, indicating that the specific deletion of YAP/TAZ in the liver not only resulted in a less severe inflammatory and fibrotic phenotype but also decelerated the progression of hepatic fibrosis induced by CCl_4_ in mice [10]. Verteporfin (VP) is a YAP inhibitor that prevents HSC activation and fibrosis in vivo [5]; however, its mechanism of action has been questioned in a recent study that covered the last 5 years [11]. All these findings necessitate the development of novel YAP-specific strategies for the amelioration of liver fibrosis.

Natural products obtained from traditional Chinese medicines exhibit significant potential in combating liver fibrosis [12–16]. *Panax japonicus* (*Panax japonicus*. C.A. Mey), i.e., the so-called *Panax japonicus* and *Panax notoginseng*, combines the efficacy of ginseng and *Panax notoginseng*. C.A. Mey is the rhizome and fleshy root of *Panax japonicus*. Chikusetsusaponin IVa (CS-IVa), derived from *P. japonicus*, is a prominent saponin compound known for its significant medicinal properties [17, 18]. Saponin extract of Panax japonicus suppresses hepatocyte EMT and HSC activation in vitro and CCl_4_-provoked liver fibrosis in mice [19]. CS-IVa attenuates isoprenaline-induced myocardial fibrosis by activating AMPK/mTOR/ULK1 signaling-mediated autophagy [17]. However, the efficacy and mechanisms of CS-IVa action in hepatic fibrosis have not been well explored. This study provides the first evidence that CS-IVa inhibits the YAP signaling pathway in vivo and in vitro to resist hepatic fibrosis. CS-IVa treatment decreased the protein expression of YAP and TAZ, consequently modulating the downstream target genes. Furthermore, direct binding of CS to YAP was confirmed by molecular docking, surface plasmon resonanc (SPR), and cellular thermal shift assay (CETSA). These findings suggest that CS-IVa is a promising therapeutic option for hepatic fibrosis.

## Materials and methods

### Materials

**Fig. 1:**
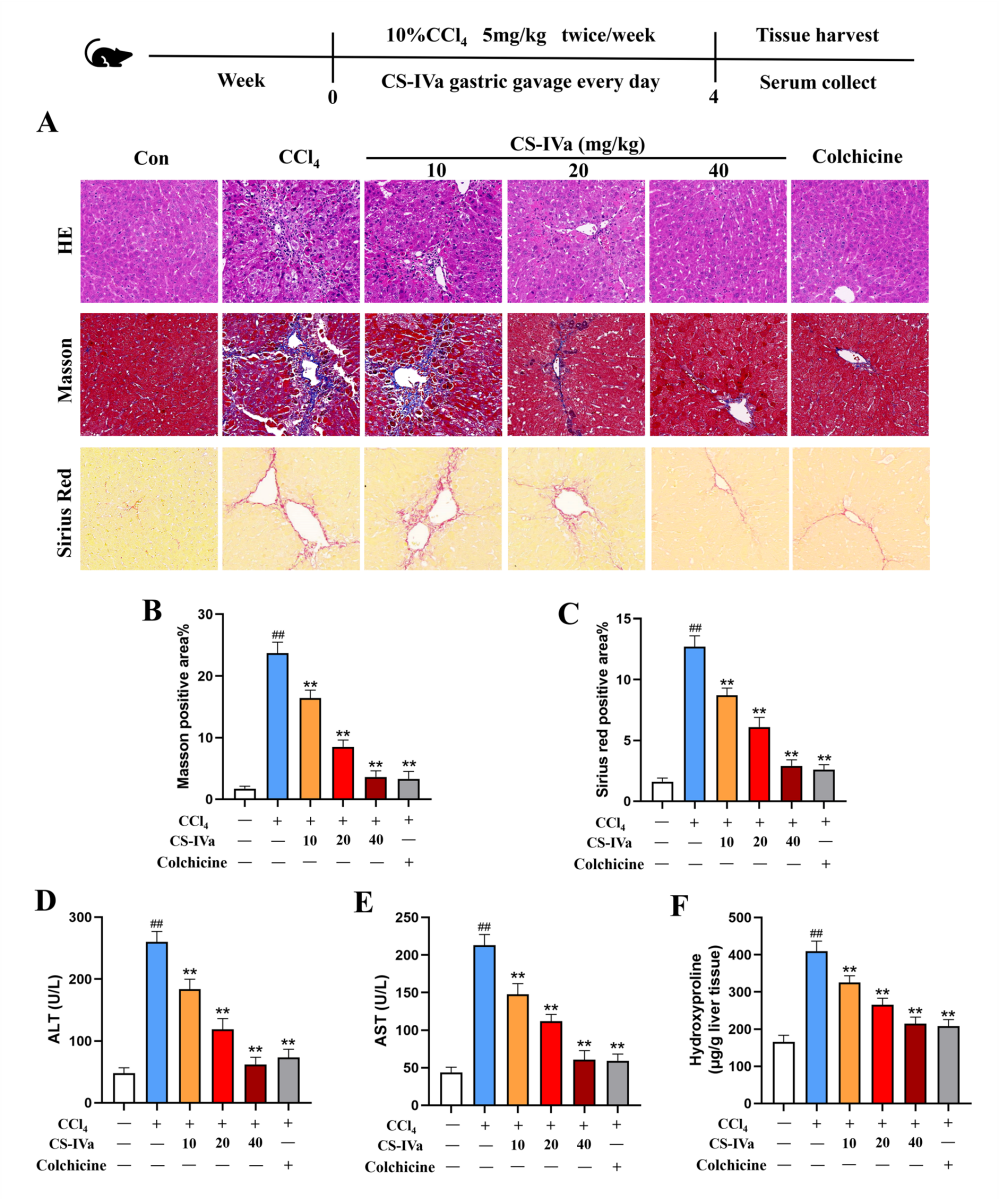
CS-IVa ameliorates CCl_4_-induced hepatic injury and hepatic fibrosis. **A** Liver histopathological alterations determined by H&E staining in mice induced with CCl_4_ and treated with or without CS-IVa; Masson's trichome and Sirius Red staining were performed on liver tissues obtained from different experimental groups; scale bar = 100 µm, n = 3. **B**, **C** Masson's trichrome- and Sirius Red-positive areas. **D**, **E** Serum levels of ALT, AST and **F** liver hydroxyproline in CCl_4_ treated mice, n = 6. The data are presented as the means ± SDs. ^##^*p* < 0.01 vs. the control group; ^**^p < 0.01 vs. the CCl_4_ group

CS-IVa (HPLC ≥ 98%) was obtained from Baoji Chenguang Biotechnology Co., Ltd. (Baoji, China). Its structure is shown in Fig. 1A. CCK-8 and VP were obtained from TOPSCIENCE Biotechnology (Shanghai, China). Colchicine was purchased from MedChemExpress Co., Ltd. (NJ). Antibodies against COL1A1 (72026S) and YAP (14074 T) were obtained from Cell Signaling Technology, Inc. (Danvers, MA). Antibodies against α-SMA (14–9760-82) were obtained from Thermo Fisher Scientific, Inc. (Shanghai, China). TAZ (ab307148) was purchased from Abcam (Cambridge, UK). Antibodies against GAPDH (41549) were purchased from Signalway Antibody, LLC (Maryland). Transforming growth factor β1 (TGF-β1) was provided by Chamot Biotechnology Co., Ltd. (Shanghai, China).

### Animals and liver fibrosis model

C57BL/6 mice (5 weeks, male, 18–20 g; Animal Research Center of Air Force Medical University, Xi’an, China) were intraperitoneally injected with CCl_4_ (10% in olive oil, twice a week) to induce liver fibrosis. The animals were randomly divided into control, CCl_4_, and CS-IVa groups and treated with CS-IVa (10, 20, or 40 mg/kg) by oral gavage daily during CCl_4_ treatment. Colchicine (0.1 mg/kg) was the positive control group. After completion of the experiment, serum and liver samples were collected. A bile duct ligation (BDL)-induced liver fibrosis model was established according to previous studies [20]. C57BL/6 mice were subjected to BDL surgery to induce hepatic fibrosis. Surgical procedures were conducted in accordance with the protocols described in the relevant literature [21]. The bile ducts were not ligated in the sham group. Mice in the CS-IVa group were intragastrically administered CS-IVa daily for 14 days after BDL. All animal experiments were approved by the Animal Care and Use Committee of Air Force Medical University (No: KY20230423).

### Proteomics analysis

#### Protein extraction and digestion

SDT (4% SDS, 100 mM Tris–HCl, pH 7.6) buffer was used to lyse the samples and extract proteins.

Protein quantification was performed using the BCA Protein Assay Kit (Beyotime). The filter-aided proteome preparation method was used to enzymatically digest an appropriate quantity of protein from each sample. After desalting on C18 cartridges (Empore™ SPE Cartridges C18 (standard density), bed I.D. 7 mm, volume 3 ml, Sigma), peptides from each sample underwent vacuum centrifugation, followed by reconstitution in 40 µl of 0.1% (v/v) formic acid (FA). Estimation of the peptide content was conducted by UV light spectral density at 280 nm using an extinction coefficient of 1.1 of 0.1% (g/l) solution calculated taking into account tryptophan and tyrosine frequency in vertebrate proteins.

#### LC–MS/MS analysis

Evosep One system liquid chromatography (Denmark) coupled with a timsTOF Pro mass spectrometer (Bruker) was used for LC–MS/MS analysis. Peptides were loaded onto a C18-reversed phase analytical column (length: 15 cm, inner diameter: 150 μm, 1.9 μm resin) in buffer A (0.1% FA in water). Buffer B (99.9% acetonitrile and 0.1% FA) was used for peptide separation at a 220 nL/min flow rate. The mass spectrometer was operated in the positive ion mode by applying an electrospray voltage of 1.6 kV. The precursors and fragments were analyzed using a TOF detector (mass range: m/z 100–1700). The timsTOF Pro spectrometer worked under a parallel accumulation serial fragmentation (PASEF) mode, collecting data considering the parameters including ion mobility coefficient (1/K0) in the range of 0.75 to 1.35 Vs cm2; 1 MS and 8 MS/MS PASEF scans. The timsTOF Pro spectrometer also actively excluded irrelevant data, and the release time was 24 s.

### Serum alanine aminotransferase (ALT)/aspartate aminotransferase (AST), total bilirubin (TBiL), and direct bilirubin (DBiL) levels and liver hydroxyproline assays

Appropriate reagent kits (Nanjing Jiancheng Institute of Biotechnology, Nanjing, China) were used to measure serum ALT, AST, TBiL, and DBiL levels. Hydroxyproline levels in the liver were determined using kits from the same company.

### Cell cultivation and intervention

LX-2 cells (Wuhan Pricella Biotechnology Co., Ltd., Wuhan, China) were seeded into 6-well plates and used for the experiments. The cells were cultured in DMEM containing 20% FBS and 100 U/ml penicillin–streptomycin solution, followed by incubation at 37 °C with 5% CO_2_. After growing to 70–80% confluence, LX-2 cells underwent 6 h of incubation in serum-free medium, followed by 24 h of treatment using 10 ng/ml TGF-β1 and CS-IVa (10, 20, and 40 μM).

### Cell transfection and inhibitors

LX-2 cells were transfected with YAP siRNA using Lipofectamine2000 (Thermo Fisher Scientific). The sequence for YAP siRNA is 5′-GACAUCUUCUGGUCAGAGA-3′. The YAP inhibitor VP was purchased from Topscience Biotechnology (Shanghai, China).

### Cell viability assay

Cells were seeded into 96-well plates (1 × 10^5^ cells/mL) at a density of 70% to 80%, and the plates were then treated with different concentrations of CS-IVa (5, 10, 20, 40, or 80 μM). CCK-8 experiments were conducted after 24 h. The results are shown in Fig. 6A.

### Quantitative real-time PCR

Liver tissues were subjected to RNA extraction using the TRIzol reagent. SYBR Green reagent was used for quantitative PCR in triplicates. mRNA expression was detected using a real-time PCR system, specifically Light Cycler 480 II (Roche, Basel, Switzerland). Data were normalized to the expression of GAPDH. The relevant qRT-PCR sequences are listed in Table 1.

**Table 1:** Primer sequences for qRT-PCR

Specises	Primer name	Forward primer (5′–3′)	Reverse primer (5′–3′)
Human	*α-SMA*	CCTTGTTTGGGAAGCAAGTGG	TGGAGCTGCTTCACAGGATT
	*COL1A1*	GTGCGATGACGTGATCTGTGA	CGGTGGTTTCTTGGTCGGT
	*YAP*	AGCCCAAGAACAGAAAGAACCT	TTGGACAAGTCCAGTGAGGC
	*Ctgf*	AAGAGAGGGCCCACGTGTA	GAAGGAGCTGTCTGTTCCACA
	*Ankrd1*	TTCGAGGCACAAGGCACAA	CCATCATTTCACTGGCGAGC
	*GAPDH*	GACCTGCCGTCTAGAAAAAC	TTGAAGTCAGAGGAGACCAC
Mouse	*α-SMA*	CCCAGACATCAGGGAGTAATGG	TCTATCGGATACTTCAGCGTCA
	*Col1a1*	TAAGGGTCCCCAATGGTGAGA	GGGTCCCTCGACTCCTACAT
	*TIMP1*	GCAACTCGGACCTGGTCATAA	CGGCCCGTGATGAGAAACT
	*GAPDH*	TGCGACTTCAACAGCAACTC	ATGTAGGCAATGAGGTCCAC

### Histopathology of liver tissue

Liver tissues were fixed in 4% paraformaldehyde for 48 h before dehydration, embedded in paraffin, and sectioning. The tissue sections were subjected to dewaxing, hydration, and staining using standard methods; H&E staining was used to evaluate hepatic morphology, and Sirius Red and Masson's trichrome staining were used to evaluate liver fibrosis. In addition, automated software for image analysis was used to quantify the infiltration of inflammatory cells and degenerative hepatocytes, and the results were expressed as the ratio of cells per 1000 hepatocytes to cells per square millimeter of liver parenchyma.

### Cell migration assays

Transwell assays were used to evaluate migration, seeding LX2 cells (2 × 10^5^ cells/200 µL) in 5% serum top chamber by virtue of a 24-well polycarbonate transwell filter (8 µm pore size, Corning Incorporated), and the lower chamber of the Transwell device was full of 20% FBS (700 µL). After 24 h of incubation, cells were fixed with 4% paraformaldehyde and stained with 0.1% crystal violet. A cotton swab was used to wipe the cells from the apical chamber. An inverted microscope was used to capture images of the migrated cells.

### Western blotting assay

Liver tissues and LX-2 cells were lysed on ice in RIPA lysis buffer supplemented with 1% phenylmethanesulfonyl fluoride. A BCA kit was used to quantify the protein concentration. To investigate the protein expression levels, western blotting was performed following standard procedures. The membranes underwent one night of incubation using antibodies against α-SMA (1:1000), COL1A1 (1:1000), YAP (1:1000), TAZ (1:1000), and GAPDH (1:5000) at 4 ℃. After three washes, the membranes were incubated with secondary antibodies (1:5000) for 1 h at room temperature. The Bradford assay (Bio-Rad) ChemiDoc™ XRS system was employed for protein band detection, and ImageJ software (version 1.53, USA) was used for the relevant analysis.

### Molecular docking

Dockey (version 1.0.3), an integrated tool for large-scale molecular docking, was used for molecular docking between CS-IVa and YAP. In brief, the PDB ID of YAP and the PubChem ClD of CS-IVa were imported into Dockey to obtain the receptor and ligand files, respectively, and the protein pocket was selected to Draw Bounding Box, and molecular docking was performed using AutoDock Vina (version number: 1.2.5). Visualization of CS-IVa and YAP complex was performed using Pymol (version number: 3.0.5) software.

### CETSA

CETSA experiments were conducted in accordance with an established protocol [22]. LX-2 cells were incubated for 24 h with CS-IVa (10 μmol/L) or DMSO, followed by lysis using lysates containing 1% protease and phosphatase inhibitors. The soluble proteins in the supernatant were isolated after 15 min of centrifugation at 12,000 rpm at 4 °C. Subsequently, the samples treated with CS-IVa or DMSO were separated into seven equal portions and heated at 45, 50, 55, 60, 65, 70, and 75 ℃, respectively, for 3 min each. The supernatants were analyzed by western blotting.

### Target validation by SPR analysis

We conducted SPR analysis to verify the targeting of CS-IVa over YAP, by diluting CS-IVa to 0.78125, 3.125, 12.5, 50 and 200 μM sequentially using a 5% DMSO PBS-P buffer solution. The flow rate was set at 30 μl/min, with binding and dissociation times of 60 and 120 s, respectively. Finally, we fitted the affinity curve using the Biacore T200 evaluation software.

### Statistical analysis

Data are presented as mean ± standard deviation. All statistical analyses were performed using GraphPad Prism 9.4. ANOVA with Bonferroni correction was used for multigroup comparisons. Statistical significance was set at P < 0.05.

## Results

### CS-IVa reversed CCl_4_-induced hepatic injury and fibrosis

To investigate the potential therapeutic effects of CS-IVa in treating hepatic fibrosis, we established a murine model of liver fibrosis via an intraperitoneal injection of CCl_4_. Four weeks of CCl_4_ administration resulted in an obvious enhancement of inflammatory cell infiltration and fibrosis in mouse livers. However, daily intragastric administration of CS-IVa (10, 20, and 40 mg/kg) ameliorated these pathological changes. Masson’s trichrome-stained tissue suggested that CS-IVa reduced the deposition of fibrous collagen and that bridging fibrosis occurred, as did Sirius Red-stained liver tissue sections (Fig. 1A–C). The efficacy of 40 mg/kg CS-IVa was similar to that of colchicine. Compared with mice treated with CCl_4_, the CS-IVa group presented significantly lower serum ALT and AST levels, suggesting that CS-IVa improved liver injury (Fig. 1D, E). Furthermore, CS-IVa significantly reduced hepatic hydroxyproline content (Fig. 1F).

### CS-IVa improved fibrogenesis induced by BDL-induced liver injury

**Fig. 2:**
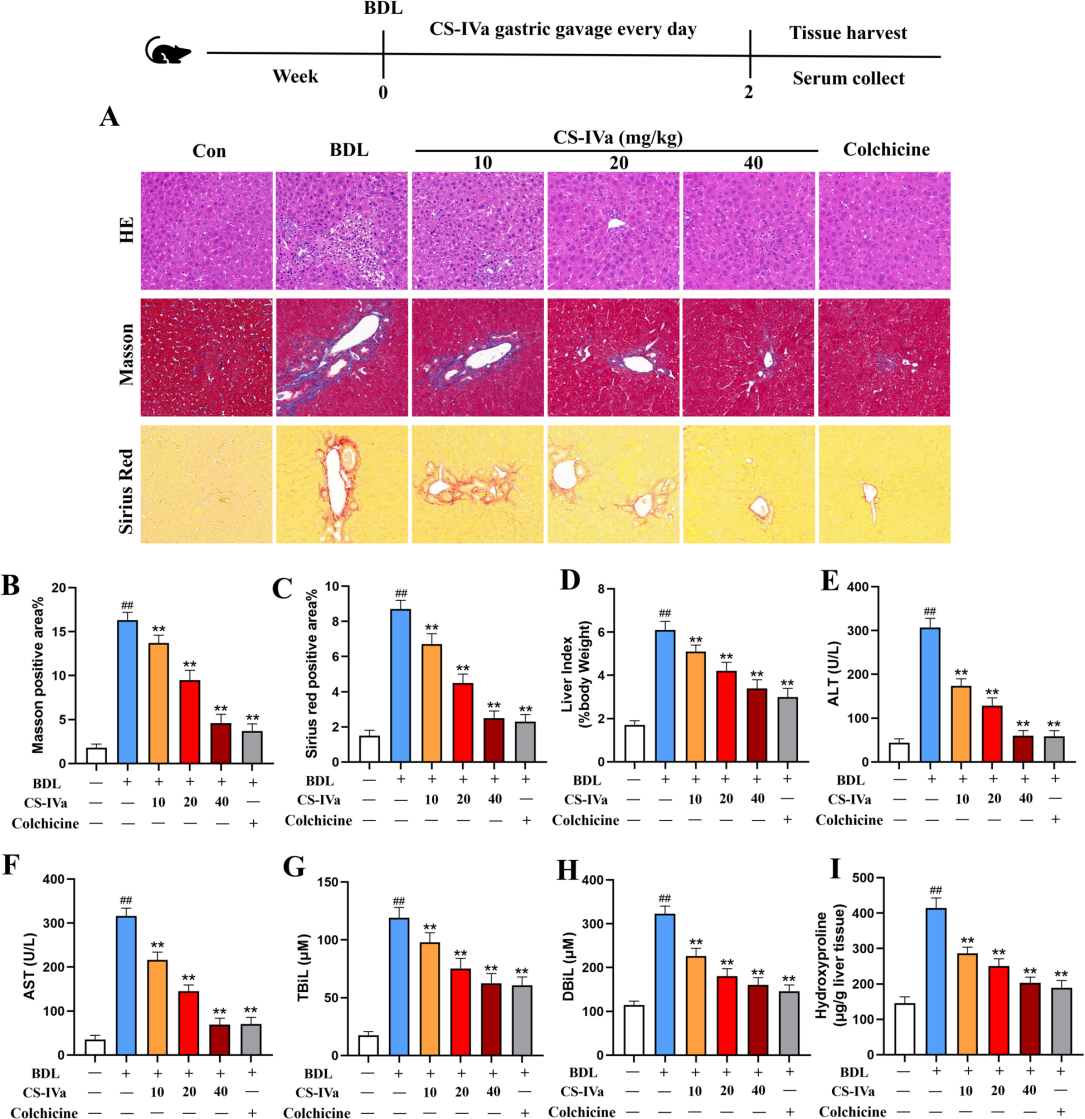
CS-IVa ameliorated BDL-induced liver injury and hepatic fibrosis. **A** Liver histopathological alterations in H&E-stained sections obtained from mice subjected to BDL and treated with or without CS-IVa; Masson's trichome and Sirius Red staining were performed on liver tissues obtained from different groups; scale bar = 100 µm, n = 3. **B**, **C** Masson's trichrome- and Sirius Red-positive areas. **D** The liver index of the different groups. **E**–**H** Serum levels of ALT, AST, TBiL, DBiL and **I** liver hydroxyproline in BDL induced mice, n = 6. The data are presented as means ± SDs. ^##^*p* < 0.01 vs. the control group; ^**^p < 0.01 vs. the BDL group

To further investigate the therapeutic effects of CS-IVa in hepatic fibrosis, we established a BDL mouse model to induce liver fibrosis. The animals were orally administered varying concentrations of CS-IVa daily for a continuous period of 14 days after common BDL. H&E staining revealed significant impairment in the morphological architecture of the hepatic tissue in the model group, accompanied by a notable increase in hepatocyte degeneration and inflammatory infiltration. In contrast, CS-IVa treatment significantly ameliorated liver injury. Masson’s trichrome and Sirius Red staining showed that CS-IVa markedly weakened the collagen matrix and fiber deposition (Fig. 2A–C). As expected, CS-IVa treatment caused a remarkable reduction in the liver index and serum ALT, AST, TBiL, and DBiL levels (Fig. 2D–H). Hydroxyproline content in the liver tissue (Fig. 2I).

### Bioinformatics analysis of proteomic data

**Fig. 3:**
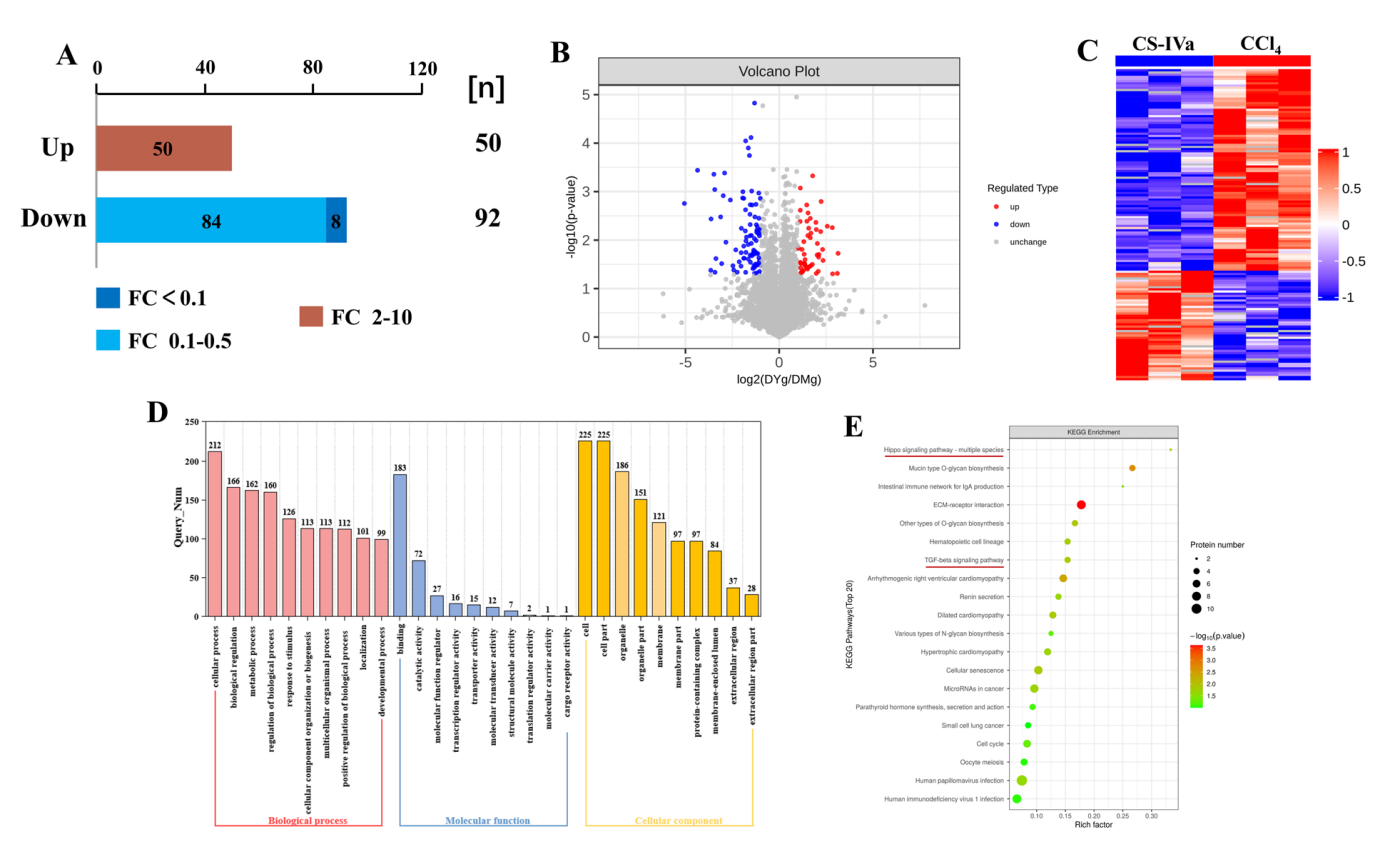
The results of proteomics. **A** The number of DEGs in CCl_4_ + CS-IVa group vs. CCl_4_ group. **B** Volcano plot of DEPs in CCl_4_ + CS-IVa group vs. CCl_4_ group. **C** Heatmap of DEPs between CCl_4_ + CS-IVa group and CCl_4_ group. **D** GO enrichment analysis of DEPs. **E **KEGG pathway enrichment analysis of DEPs

Mice with liver fibrosis were subjected to liver proteomics experiments, and the protective effect of CS-IVa on liver fibrosis was examined. A total of 148 differentially expressed proteins (DEPs), including 88 upregulated and 60 downregulated proteins, were identified by comparing the CCl_4_ group with the control group (Fig. S1A and Fig. S1B). Meanwhile, a total of 142 DEPs, including 50 upregulated proteins and 92 downregulated proteins, were identified by comparison of CS-IV group with the CCl_4_ group (Fig. 3A, B). KEGG enrichment analysis was performed to investigate the functions of the above filtrated DEPs. As shown in Fig. S1E and Fig. 3E, the DEPs in the CCl_4_ compared with the control group and the DEPs in the CS-IV group compared with the CCl_4_ group were almost always enriched in the Hippo and TGF-β signaling pathways, suggesting that the effect of CS-IVa on fibrosis is closely relevant to these two pathways.

### CS-IVa repressed the YAP pathway in the liver tissue

**Fig. 4:**
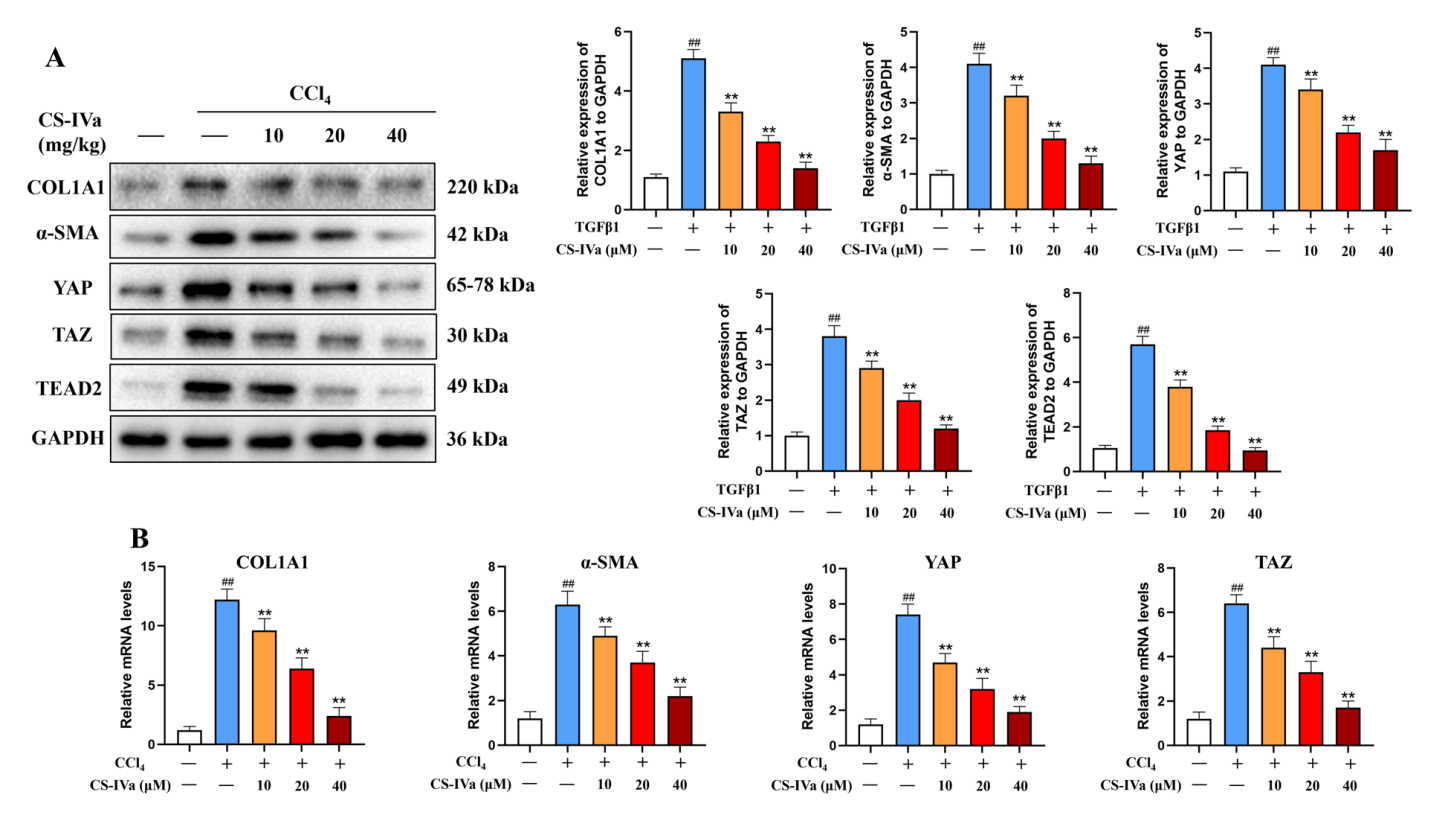
CS-IVa inhibited hepatic fibrosis induced by CCl_4_ through downregulating the YAP pathway. **A** CS-IVa treatment led to a significant decrease in the protein expression of YAP, TAZ, TEAD2, α-SMA and COL1A1 in liver tissues affected by CCl_4_. **B** The mRNA levels of *YAP*, *TAZ*, *α-SMA* and *COL1A1* also downregulated by CS-IVa, n = 3. The data are presented as means ± SDs. ^##^*p* < 0.01 vs. the control group; ^**^p < 0.01 vs. the CCl_4_ group

**Fig. 5:**
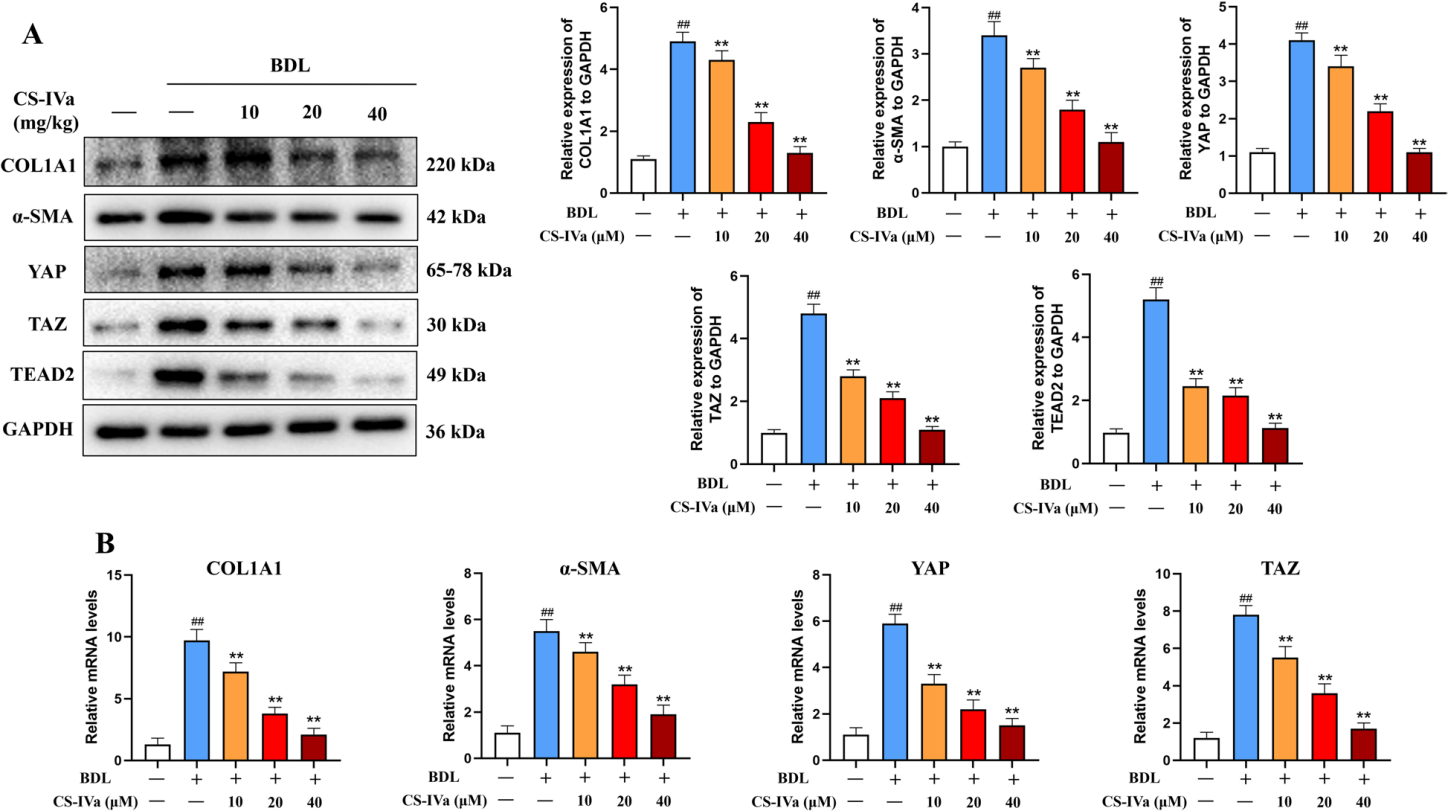
CS-IVa inhibited liver fibrosis induced by BDL through inhibition of the Hippo pathway. **A** The protein expression of YAP, TAZ, TEAD2, α-SMA and COL1A1 were decreased by CS-IVa in mouse liver tissues. **B** The mRNA levels of *YAP*, *TAZ*, *α-SMA* and *COL1A1* were assessed in liver samples obtained from mice, CS-IVa obviously decreased the mRNA expression of these genes, n = 3. The data are presented as means ± SDs. ^##^*p* < 0.01 vs. the control group; ^**^p < 0.01 vs. the BDL group

A substantial body of research has established the critical role of the Hippo/YAP pathway in liver fibrosis, wherein the activation and inhibition of YAP act as pivotal regulators in the development and progression of this condition. The unphosphorylated form of YAP can translocate from the cytoplasm to the nucleus, where it interacts with multiple transcription factors, including TEAD, to form stable complexes. The levels of YAP, TAZ and TEAD2 were elevated in mice with CCl_4_- and BDL-induced fibrosis (Figs. 4A and 5A), which was reversed by treatment with CS-IVa. Additionally, the expression of α-SMA and COL1A1 exhibited analogous findings. qRT-PCR demonstrated an obvious reduction in the mRNA expression of *YAP*, *TAZ*, *α-SMA*, and *COL1A1* genes following CS-IVa treatment (Figs. 4B and 5B). These findings indicated that CS-IVa mitigated CCl_4_- and BDL-induced hepatic fibrosis by downregulating the YAP/TAZ pathway.

### CS-IVa repressed TGF-β1 induced HSC activation via the YAP pathway

**Fig. 6:**
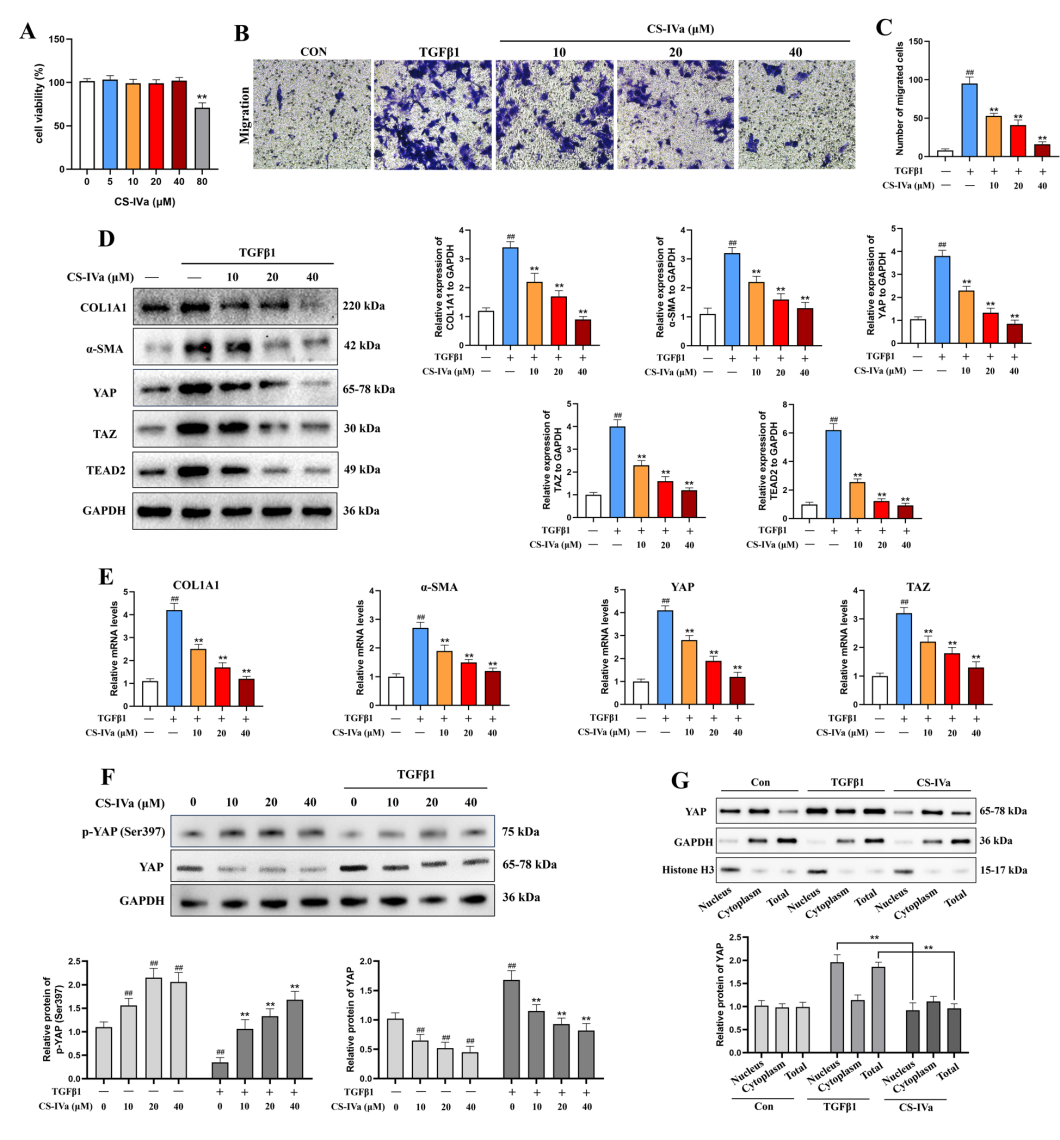
CS-IVa represses TGF-β1 induced HSC activation through the Hippo pathway. **A** The cell viability of LX-2 treated by CS-IVa. **B** cell migration was analyzed by transwell assay (scale bar: 50 μm). **C** The number of migrated cells. **D** The protein expression levels of YAP, TAZ, TEAD2, α-SMA and COL1A1 were markedly decreased by CS-IVa in LX-2 cells. **E** CS-IVa downregulated the mRNA expression of *YAP*, *TAZ*, *α-SMA* and *COL1A1*. **F** The protein expression level of p-YAP (Ser 397). **G** Protein expression of YAP in nucleus and cytoplasm. The data are presented as means ± SDs. ^##^*p* < 0.01 vs. the control group; ^**^p < 0.01 vs. the BDL group

The upregulated migration f LX-2 HSCs in response to TGF-β was dose-dependently inhibited by CS-IVa (Fig. 6B, C). The YAP signaling pathway has a significant effect on HSC activation. The initial phase of HSC activation involves the translocation of YAP into the nucleus to interact with various transcription factors, thereby increasing the expression level of subsequent target genes. Activation of the YAP/TAZ pathway can trigger fibrosis in the liver tissues by controlling myofibroblast activation. To determine the suppressive effect of CS-IVa on HSC activation via the YAP/TAZ pathway, we stimulated LX-2 cells with TGF-β1 by applying varying concentrations of CS-IVa. TGF-β1 treatment of LX-2 cells markedly elevated YAP, TAZ and TEAD2 levels. After treatment with CS-IVa, there was a notable reduction in the mRNA expression of *COL1A1*, *α-SMA*, *YAP* and *TAZ*, and protein expression of COL1A1, α-SMA, YAP, TAZ and TEAD2 (Fig. 6D, E).

As shown in Fig. 6F, we found that CS-IVa treatment increased YAP phosphorylation at Ser397, and the phosphorylation of YAP at Ser-397 results in YAP protein degradation. Research has demonstrated that the nuclear translocation of YAP is a critical determinant of its efficacy. Furthermore, YAP expression was identified following nuclear protein extraction. CS-IVa not only downregulated YAP protein expression but also effectively inhibited its nuclear translocation (Fig. 6G).

**Fig. 7:**
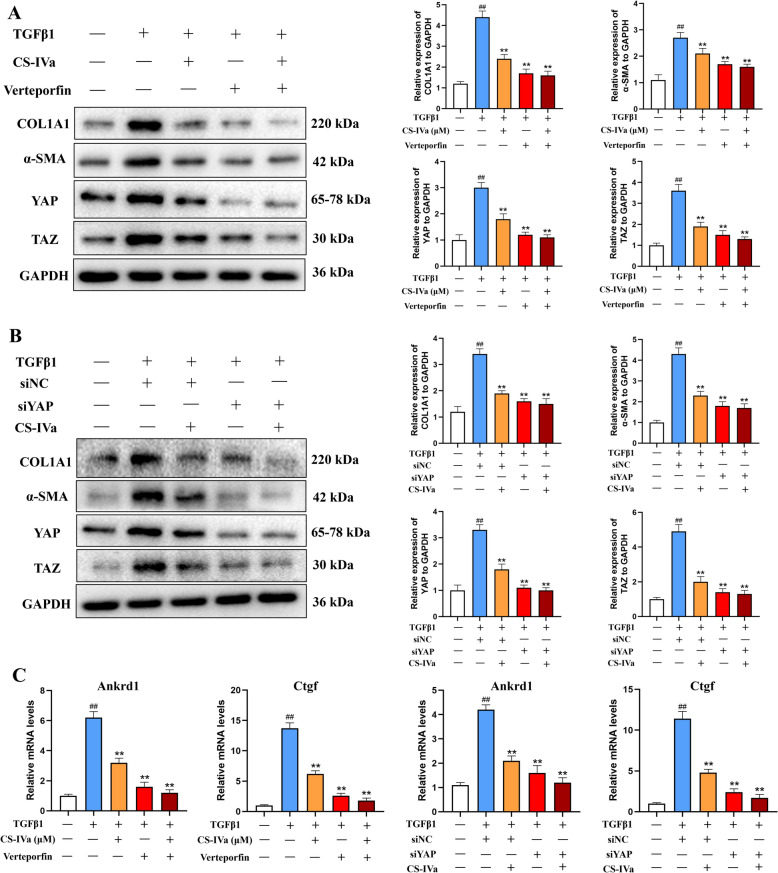
CS-IVa mediated inhibition of HSC activation depends on regulation of the Hippo pathway. **A** LX-2 cells were treated with CS-IVa (40 μM) and/or verteporfin, and COL1A1, α-SMA, YAP and TAZ protein expression was detected via western blot analysis. **B** YAP siRNA and CS-IVa (40 μM) were used to treat LX-2 cells, followed by WB analysis of the protein expression of COL1A1, α-SMA, YAP and TAZ. **C** LX-2 cells were treated with CS-IVa (40 μM), either alone or in combination with YAP siRNA and verteporfin. Then, the mRNA levels of *Ctgf* and *Ankrd1* were measured by RT‒qPCR (n = 3), The data are presented as means ± SDs. ^##^*p* < 0.01 vs. the control group; ^**^p < 0.01 vs. the BDL group

To investigate the potential role of the YAP/TAZ pathway in CS-IVa-induced repression of hepatic fibrosis, we treated LX-2 cells with VP in combination with CS-IVa. As shown in Fig. 7A, VP (1 μM) significantly inhibited TGF-β1-induced YAP, TAZ, α-SMA, and COL1A1 expression. However, CS-IVa-VP co-treatment failed to lower the levels of these proteins. To investigate the effect of YAP on the suppression HSC activation, we conducted YAP knockdown experiments in LX-2 cells. YAP knockdown markedly inhibited YAP, TAZ, α-SMA, and COL1A1 expression, whereas α-SMA and COL1A1 expression did not decrease after CS-IVa treatment (Fig. 7B). In addition, CS-IVa treatment led to an obvious reduction in the levels of *Ctgf* and *Ankrd1*, which are downstream target genes of YAP, in LX-2 cells. The knockdown or inhibition of YAP did not result in further reduction of YAP levels or its downstream target genes upon CS-IVa treatment (Fig. 7C). In summary, these findings suggest that CS-IVa mitigates HSC activation and liver fibrosis by modulating the YAP/TAZ signaling pathway.

### YAP is the key target of CS-IVa in preventing liver fibrosis

**Fig. 8:**
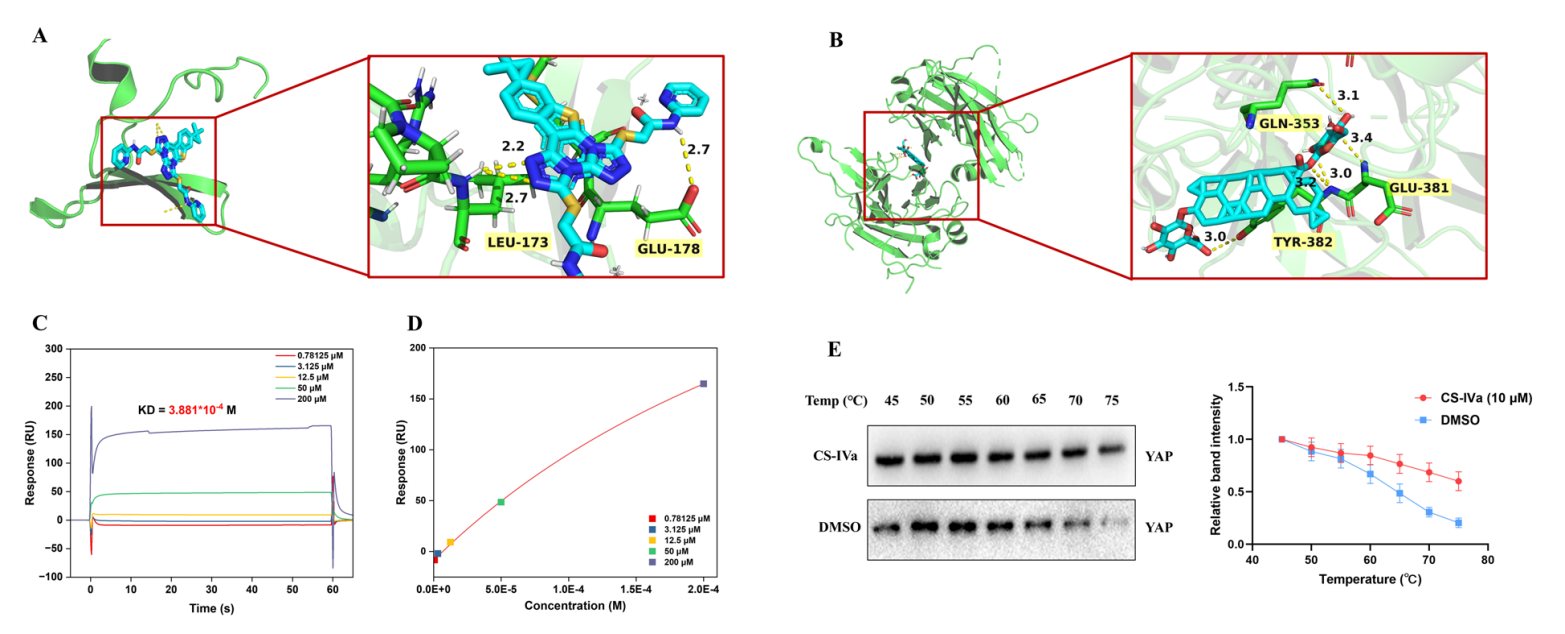
YAP is the key target of CS-IVa. **A** The schematic diagram of the molecular docking of CS-IVa with YAP, shown as the 3D diagram. The ribbon and stick structure displays the predicted bonds between CS-IVa and YAP. **B** The schematic diagram of the molecular docking of CS-IVa with TEAD2, shown as the 3D diagram. The ribbon and stick structure displays the predicted bonds between CS-IVa and TEAD2. **C** Response value of interaction between CS-IVa and YAP on CM5 chip. **D** Affinity fitting curves corresponding to reporting points of gradient concentrations. **E** The stability of the YAP protein was evaluated by CETSA at temperatures ranging from 45 to 75 °C in the presence and absence of CS-IVa

Molecular docking, SPR, and CETSA experiments were used to further confirm the binding between CS-IVa and YAP according the published studies [23, 24]. Molecular docking revealed that CS-IVa was capable of forming hydrogen bonds with the residues LEU-173 and GLU-178 of YAP. The binding energy of −11.1 ± 0.03 kcal/mol (Fig. 8A) verified that CS-IVa bound well to YAP. Simultaneously, CS-IVa was capable of forming hydrogen bonds with the residues GLN-353, TYR-382 and GLU-381 of TEAD2. The binding energy of −15.26 ± 0.03 kcal/mol verified that CS-IVa bound well to TEAD2 (Fig. 8B). The experimenters fixed YAP in the chip, allowing the CS-IVa solution at five concentrations to flow through the chip, aiming to examine the binding energy in the SPR detection. YAP bound to CS-IVa with a dissociation constant of 3.88 × 10^−4^ M (Fig. 8C, D). CETSA was used to further validate the binding of CS-IVa to YAP. As shown in Fig. 8E, treatment with CS-IVa significantly increased the thermal stability of YAP, indicating that YAP may directly benefit from the antifibrotic effects of CS-IVa.

## Discussion

Liver fibrosis arises from persistent inflammation that accompanies different liver diseases and is characterized by alterations in the ECM, including increased collagen and fibrin deposition, as well as changes in its composition, and in severe cases, it can culminate in cirrhosis and hepatocellular carcinoma [25]. Considering the limited therapeutic strategies specific to liver fibrosis, it is imperative to develop novel pharmaceutical interventions to manage this condition. Inhibiting HSC activation serves as a potential therapeutic approach for treating liver fibrosis. The intricate and diverse chemical structures of natural products have demonstrated significant potential for combating liver fibrosis, and these products are promising candidates for novel drug development [26–30].

In this study, two animal models were used to evaluate the anti-hepatic fibrosis effect of CS-IVa. The carbon tetrachloride (CCl₄)-induced liver fibrosis model and the bile duct ligation (BDL)-induced liver fibrosis model are two widely utilized experimental animal models for investigating the mechanisms and therapeutic approaches of liver fibrosis. Each model possesses distinct advantages and limitations, rendering them appropriate for diverse research objectives. CCl₄-induced liver injury and fibrosis exhibit characteristics similar to those observed in human alcoholic liver disease (ALD) and non-alcoholic steatohepatitis (NASH) [31]. This model induces liver fibrosis via intraperitoneal injection or oral administration, eliminating the need for surgery and reducing technical complexity. Moreover, the extent of fibrosis can be precisely regulated by modulating the dose and frequency of administration. However, a disadvantage of the CCl₄ model is that CCl₄ not only induces hepatic injury but also has the potential to cause toxicity in other organs, such as the kidneys. BDL can induce significant liver fibrosis and cholestasis within a short period, making it an appropriate model for studying acute liver injury and cholestatic liver disease [32]. However, this model is not suitable for investigating other types of liver fibrosis, such as those caused by alcoholic or metabolic factors. BDL necessitates a surgical procedure that is technically challenging, resulting in high postoperative mortality rates among animals. Additionally, potential surgical complications, such as infection and hemorrhage, may compromise the experimental outcomes.

According to previous studies, the activation or senescence of HSCs is significantly influenced by YAP, which regulates their fibrotic activity through the precise control of this transitional state. HSC-specific knockdown of YAP induces cellular senescence and confers protection against liver fibrosis [8]. The upregulation of YAP can prolong its activation and enhance ECM synthesis, thereby exacerbating fibrosis. Upon activation and nuclear translocation, YAP promotes the transcriptional activation of *Ctgf* and *Ankrd1*, driving HSC activation. Furthermore, the upregulation of *Ctgf* expression has been observed in hepatic fibrosis and activated HSCs, facilitating the synthesis and secretion of ECM proteins. VP is a YAP inhibitor and can restrict the YAP-TEAD activity, thereby promisingly preventing development of cancers [33–35]. Nevertheless, research has not confirmed its antifibrotic activity but has only highlighted the large promoting effect of YAP and TAZ on liver fibrosis. The utilization of natural components derived from traditional Chinese medicines shows promising potential in the treatment of liver fibrosis. CS-IVa crucially constitutes the Rhizoma *Panacis japonica*. However, the efficacy and mechanism of its anti-hepatic fibrotic activity remain unclear. In our study, the antifibrotic effects of CS-IVa were validated in two animal models of hepatic fibrosis, thereby elucidating the pharmacological mechanisms of CS-IVa. Our study showed that CS-IVa exerted potent antifibrotic effects by suppressing YAP/TAZ protein expression in vitro and in vivo. Molecular docking, SPR, and CETSA showed that CS-IVa could directly bind to YAP.

Further mechanistic investigation revealed that YAP knockdown did not augment the inhibitory effects of CS-IVa on HSC activation or fibrosis-related protein expression. VP attenuated YAP and TAZ levels following TGF-β1 stimulation and reduced fibrosis markers, suggesting its potential as a therapeutic agent for liver fibrosis [36]. However, VP did not augment the suppressive effect of CS-IVa on HSC activation or fibrosis-related protein expression. *Ctgf* and *Ankrd1* were expressed in a similar manner. Furthermore, molecular docking, SPR, and CETSA experiments confirmed that YAP was a direct target of CS-IVa. Collectively, our study suggests that the antifibrotic effect of CS-IVa may be mediated through targeted inhibition of the YAP/TAZ signaling pathway. However, this study had several limitations, including the need to address the efficacy of CS-IVa in human administration, its long-term safety profile, and the determination of its pharmacokinetic parameters. In future studies, we intend to enhance the efficacy of CS-IVa through structural modifications and further explore its pharmacokinetic parameters.

## Conclusion

In conclusion, our findings provide strong evidence supporting the significant antifibrotic effects of CS-IVa by effectively suppressing HSC activation both in vitro and in vivo. Direct binding of CS-IVa to YAP suggests its potential as a YAP inhibitor. Treatment with CS-IVa effectively inhibited YAP and TAZ levels in the Hippo pathway, consequently inhibiting downstream target gene expression. This study confirmed the effectiveness of CS-IVa in reducing liver fibrosis and elucidated the underlying mechanism through which HSC activation and liver fibrosis were effectively suppressed by CS-IVa. Hence, CS-IVa shows potential as a novel YAP inhibitor for the treatment of hepatic fibrosis.

## Data Availability

The datasets are available from the corresponding author on request.

## Abbreviations

HSCs: Hepatic stellate cells
YAP: Yes-associated protein
TAZ: Tafazzin
*Ctgf*: Connective tissue growth factor
Ankrd1: Cardiac ankyrin repeat protein
CS-IVa: Chikusetsusaponin Iva
CCK-8: Cell counting kit-8
COL1A1: Collagen alpha-1(I) chain
α-SMA: α-Smooth muscle actin
TGF-β1: Transforming growth factor β1
GAPDH: Glyceraldehyde-3-phosphate dehydrogenase
CCl_4_: Carbon tetrachloride
BDL: Bile duct ligation
ALT: Alanine aminotransferase
AST: Aspartate aminotransferase
TBiL: Total bilirubin
DBiL: Direct bilirubin
MD: Molecular dynamics
SPR: Surface plasmon resonance
CETSA: Cellular thermal shift assay
VP: Verteporfin
FA: Formic acid
PASEF: Parallel accumulation serial fragmentation
BP: Biological process
DEP: Differentially expressed protein
